# G protein-coupled receptor kinase 2 moderates recruitment of THP-1 cells to the endothelium by limiting histamine-invoked Weibel-Palade body exocytosis

**DOI:** 10.1111/jth.12470

**Published:** 2014-02-07

**Authors:** N L Stevenson, B Martin-Martin, J Freeman, J Kriston-Vizi, R Ketteler, D F Cutler

**Affiliations:** * Endothelial Cell Biology Laboratory, MRC Laboratory for Molecular Cell Biology, UCLLondon, UK; † Translational Research Resource Centre, MRC Laboratory for Molecular Cell Biology, UCLLondon, UK; ‡ Bioinformatics Image Core, MRC Laboratory for Molecular Cell Biology, UCLLondon, UK

**Keywords:** G protein coupled receptor kinase 2, human umbilical vein endothelial cells, P-selectin, von Willebrand factor, Weibel-Palade bodies

## Abstract

**Background:**

G protein-coupled receptors (GPCRs) are a major family of signaling molecules, central to the regulation of inflammatory responses. Their activation upon agonist binding is attenuated by GPCR kinases (GRKs), which desensitize the receptors through phosphorylation. G protein-coupled receptor kinase 2(GRK2) down-regulation in leukocytes has been closely linked to the progression of chronic inflammatory disorders such as rheumatoid arthritis and multiple sclerosis. Because leukocytes must interact with the endothelium to infiltrate inflamed tissues, we hypothesized that GRK2 down-regulation in endothelial cells would also be pro-inflammatory.

**Objectives:**

To determine whether GRK2 down-regulation in endothelial cells is pro-inflammatory.

**Methods:**

siRNA-mediated ablation of GRK2 in human umbilical vein endothelial cells (HUVECs) was used in analyses of the role of this kinase. Microscopic and biochemical analyses of Weibel-Palade body (WPB) formation and functioning, live cell imaging of calcium concentrations and video analyses of adhesion of monocyte-like THP-1 cells provide clear evidence of GRK2 function in histamine activation of endothelial cells.

**Results:**

G protein-coupled receptor kinase 2 depletion in HUVECs increases WPB exocytosis and P-selectin-dependent adhesion of THP-1 cells to the endothelial surface upon histamine stimulation, relative to controls. Further, live imaging of intracellular calcium concentrations reveals amplified histamine receptor signaling in GRK2-depleted cells, suggesting GRK2 moderates WPB exocytosis through receptor desensitization.

**Conclusions:**

G protein-coupled receptor kinase 2 deficiency in endothelial cells results in increased pro-inflammatory signaling and enhanced leukocyte recruitment to activated endothelial cells. The ability of GRK2 to modulate initiation of inflammatory responses in endothelial cells as well as leukocytes now places GRK2 at the apex of control of this finely balanced process.

## Introduction

To initiate an inflammatory response, both leukocytes and endothelial cells need to be activated by hormones and pro-inflammatory cytokines. The subsequent cell-surface expression of adhesion molecules in both cell types results in the rolling of leukocytes along the endothelial surface, before eventual firm adhesion and extravasation 1.

Many signaling pathways involved in both endothelial and leukocyte activation are initiated by stimulating G protein-coupled receptors (GPCRs) 2. GPCR signal transduction is attenuated by GPCR kinase (GRK)-mediated phosphorylation of agonist-bound receptor. This promotes β-arrestin binding, which uncouples the receptor from its G proteins and mediates receptor internalization and recycling 3,4. Disruption of this machinery alters the strength and/or duration of physiological responses to GPCR ligands 5. Of seven GRK subfamilies, the ubiquitously expressed GRK2 has been most closely linked to inflammatory 2,6 and cardiovascular function 6,7.

GRK2 protein levels are higher in leukocytes than other tissues 8 and its cytokine-induced down-regulation 9–11 is associated with chronic inflammatory disorders such as multiple sclerosis (MS) 12 and rheumatoid arthritis (RA) 9,13, as well as inflammatory pain 14. The pathologies of both MS and RA are characterized by increased leukocyte infiltration of diseased tissues, probably caused, at least in part, by impaired GRK2-mediated attenuation of chemokine signaling. For example, GRK2^+/−^ murine T cells show significantly heightened migratory responses towards CCL4. This is concurrent with enhanced calcium signaling and PKB phosphorylation, indicative of impaired CCR5 desensitization 15. Moreover, loss of GRK2 in the endothelium can enhance cytokine expression, increasing the incidence of macrophage extravasation in endothelial-GRK2^−/−^ mice 16.

Endothelial activation is mediated by pro-inflammatory and procoagulant factors, delivered to the endothelial cell surface by exocytosing Weibel-Palade bodies (WPBs). These specialized secretory organelles store the multimeric glycoprotein von Willebrand factor (VWF) 17 and are formed at the *trans*-Golgi network 18–20, with the help of an AP-1/clathrin coat 21. Upon injury or infection, mature organelles, previously anchored to cortical actin 22, fuse with the plasma membrane and release VWF to initiate hemostasis 23,24. Other WPB cargo such as the leukocyte receptor P-selectin, its co-factor CD63 25 and pro-inflammatory cytokines are also delivered to the cell surface or released into the circulation. WPBs are thus central to endothelial regulation of inflammation.

As GRK2-deficiency in leukocytes has been closely linked to inflammatory disorders, we determined whether it also affects the pro-inflammatory behaviour of endothelial cells.

## Materials and methods

### Cell culture and transfection

Human umbilical vein endothelial cells (HUVECs, TCS Cellworks, Buckingham, UK) and THP-1 cells (a gift from Dr Patric Turowski) were cultured as previously described 26. Two-round nucleofections (Nucleofector II, programme U-001, Amaxa Biosystems, Gaithersbrg, MD, USA) with 200 pmol siRNA and 10^6^ HUVECs (passage 3) were performed 48 h apart for assay 48 h later. GRK2 siRNA sequence 1: 5′-UGUCCAGUAACUUGAUUCC-3′ (Sigma, St Louis, WA, USA), sequence 2: 5′-GCUCGCAUCCCUUCUCGAAUU-3′. Mock transfections were performed using firefly luciferase siRNA 27: 5′-CGUACGCGGAAUACUUCG-3′. For ssHRP 28 expression, 7 μg construct was included in the second reaction.

### Antibodies

Antibodies used were: rabbit polyclonal anti-human VWF antibody and its HRP-conjugated form (DAKO, Cambridgeshire, UK); sheep polyclonal anti-TGN46 (Abcam, Cambridge, UK); anti-P-selectin (R&D Systems, Minneapolis, MN, USA); rabbit polyclonal anti-GRK2; mouse monoclonal anti-β-actin (Santa Cruz Biotechnology, Middlesex, UK); mouse monoclonal anti-MyRIP (a gift from Professor Seabra, Imperial College London); and Alexafluor-conjugated (Invitrogen, Paisley, UK) and HRP-conjugated (Jackson Immunoresearch, Suffolk, UK) secondary antibodies.

### Immunofluorescence and WPB quantification

Transfected HUVECs were fixed and stained as described previously 21. Images were taken using a Leica TCS SPE scanning confocal microscope, a 63× (NA1.3) or 40× (NA1.15) oil immersion lens (NA 1.15) and LAS-AF Software (Leica, Buckinghamshire, UK). Acquisition settings were: 0.5 μm z-stack step size, 1024 × 1024 pixel resolution, 3–4 frame average and 1× zoom. For quantification, the LAS-AF mark-and-find feature and a motorized stage were used to generate 15–25 random fields of view (FoV, 300–400 cells) per treatment condition in each replicate experiment. WPBs were quantified using Image J; background subtraction was performed on the VWF channel using a rolling ball algorithm (radius 2 pixels) and a manual threshold applied. Segmented objects over 0.1 μm^2^ were counted and measured for Feret's diameter (defined as the longest diameter across a WPB in 2D projection) using Image J.

### Secretion assays

VWF secretion assays have been described previously 21. Briefly, cells were rinsed and incubated in release medium for 30 min, then release medium plus 100 ng mL^−1^ phorbol 12-myristate 13-acetate (PMA, Sigma-Aldrich, St Louis, MO, USA) or 10 μm histamine for 45 or 30 min, respectively. Medium was collected to sample VWF release and intracellular VWF determined from cell lysates. Secreted VWF is presented as a percentage of the total VWF (media plus lysates). All results are normalized to total lysate protein content, as determined by bicinchoninic assay (Pierce, Rockford, IL, USA). Total VWF measurements were used to compare VWF protein expression in mock and GRK2-depleted cells. To measure unregulated secretion over longer periods, cells were rinsed and incubated in serum-free release medium for 4 h, or in optiMEM reduced serum medium (GIBCO, Paisley, UK) for 7 h.

### ssHRP secretion assays

Eight hours post-transfection, cells were rinsed and incubated in phenol red-free HUVEC growth medium (Sigma); 17 h later, medium was collected and cells lysed in 50 mm Tris/Cl pH2 on ice. Samples were processed and analyzed as described previously 29. Secretion is presented as a proportion of total ssHRP (media plus lysates).

### Western blotting

Cell lysates were collected in RIPA buffer (50 mm Tris-HCl pH 7.5, 300 mm NaCl, 1% deoxcholate, 2% Triton-X100, 0.1% SDS). Proteins were separated by SDS-PAGE on 8% acrylamide gels prior to transfer onto a protran nitrocellulose transfer membrane. Separation of pro- and mature VWF was performed on precast Novex® (Life Technologies, Paisley, UK) 6% Tris-Glycine 1.5 mm gels. Blots were probed with primary and HRP-conjugated secondary antibodies (above). Band quantification was performed using a Molecular Imager GS-800 densitometer and Quantity One Software (version 4.6.3, BioRad, Hertfordshire, UK), normalizing against a β-actin loading control.

### VWF multimer analysis

Samples were concentrated using Vivaspin500 centrifugal filter units (Sartorius, Goettingen, Germany) and run on SDS-agarose gels as described previously 29. Multimer patterns were analyzed using the Image J plot profile function to measure intensity changes down each lane, normalized against total signal.

### Quantitative PCR

RNA extraction, cDNA preparation and PCR were performed as described previously 22, quantifying normalized expression (using actin) by the ΔΔCT method 30. Single product amplification was verified by melting curve analysis and gel electrophoresis. Primers used were: GRK2 forward: 5′-GGACAGTGATCAGGAGCTCTA-3′; reverse: 5′-AAGGACTGCATCATGCATGGC3-'; VWF forward: 5′-GCCATCATGCATGAGGTCAGA-3′; reverse: 5′-GGCTCCGTTCTCATCACAGAT-3′; actin forward: 5′-TGGTGGTGAAGCTGTAGCC-3′; reverse: 5′-GCGAGAAGATGACCCAGAT-3′; HRH1 forward: 5′-GGGCCGTCCTCTCTGCCTCTT-3′; reverse: 5′-GCCGAGGCTCGGGTCTTGGT-3′.

### Calcium imaging

2× Fluo-4 calcium indicator, containing 25 μm probenecid, was prepared in serum-free release medium as per manufacturer's instructions (Fluo-4 Direct™ Calcium Assay Kit, Molecular Probes, Life Technologies, Paisley, UK). Nucleofected cells seeded onto 1.45-mm glass-bottom imaging dishes (PAA) were rinsed and loaded with 1× Fluo-4 for 30 min at 37 °C/5% CO_2_ prior to imaging. Movies were acquired using an UltraVIEW VoX spinning disc system (PerkinElmer, Waltham, MA, USA) mounted on an inverted microscope (TiE; Nikon, Surrey, UK) with an EM charge-coupled device camera (512 × 512 pixels; C9100-13; Hamamatsu Photonics, Hertfordshire, UK) and 488- and 561-nm solid-state lasers. Cells were visualized using a 100× oil immersion lens (NA 1.4), inside a 37 °C heat-controlled chamber. Z-stacks of 0.5-μm spacing were acquired using a piezo stage (NanoScanZ; Prior Scientific, Cambridgeshire, UK) every 10 s for 10 min. At time-points 60, 300 and 480 s, 10 mm histamine, 200 μm A21387 ionophore and 50 mm EGTA were added, respectively, to stimulate cells and provide a maximal (fmax) and minimal fluorescent signal. To determine changes in intracellular calcium levels, a 40 × 40 pixel ROI was drawn juxtanuclear in the cytosol for each cell and the mean fluo-4 intensity measured at every time-frame using Velocity software (PerkinElmer). Measurements were normalized against fmax to eliminate inconsistencies in indicator loading. To align curves for comparison, Fluo-4 intensity at time-point 60 s was subtracted from all values.

### THP-1 flow assays

Nucleofected HUVECs were seeded onto gelatin-coated μ-slides VI^0.4^ (ibidi, Munich, Germany). Slides were mounted on the microscope stage of an Axiovert 100 (Carl Zeiss, Welwyn Garden City, UK), maintained at 37 °C, and connected to a syringe pump system (Harvard Aparatus, Holliston, MA, USA) to draw fluid through the chamber with a wall shear stress of 0.07 Pa (0.7 dyne/cm^2^). Cells were rinsed with perfusion medium (HBSS containing Ca^+2^ and Mg^+2^ and 0.2% BSA) under flow, then 10^6^ THP1 cells mL^−1^ were perfused across the endothelial surface for 3 min to image steady-state rolling. Next, a 10-min stimulation of HUVECs, using perfusion medium + 10 μm histamine, was followed by a second THP-1 cell perfusion in the absence of secretagogue. The latter was recorded for 5 min to observe monocyte rolling. Movies were taken at 10× objective with a FoV of 784 × 576 pixels (559.78 × 411.26 μm) using a QIMAGING Scientific (QIMAGING Scientific, Surrey, Canada) CMOS Rolera bolt camera, acquiring at a rate of 24 frames/second, and Micro-Manager 1.4.13 software. To quantify firmly adhered monocytes on the endothelial surface, random snapshots were taken in the absence of flow at the end of the movie. In antibody blocking experiments, HUVECs were incubated with 15 μg mL^−1^ sheep polyclonal anti-human P-selectin (R&D Systems, Minneapolis, MN, USA) or IgG from sheep serum (Sigma-Aldrich) at 37 °C for 30 min prior to experimentation. Antibody was present for the remainder of the experiment.

## Results

### Loss of GRK2 reduces WPB numbers

**Figure 1:**
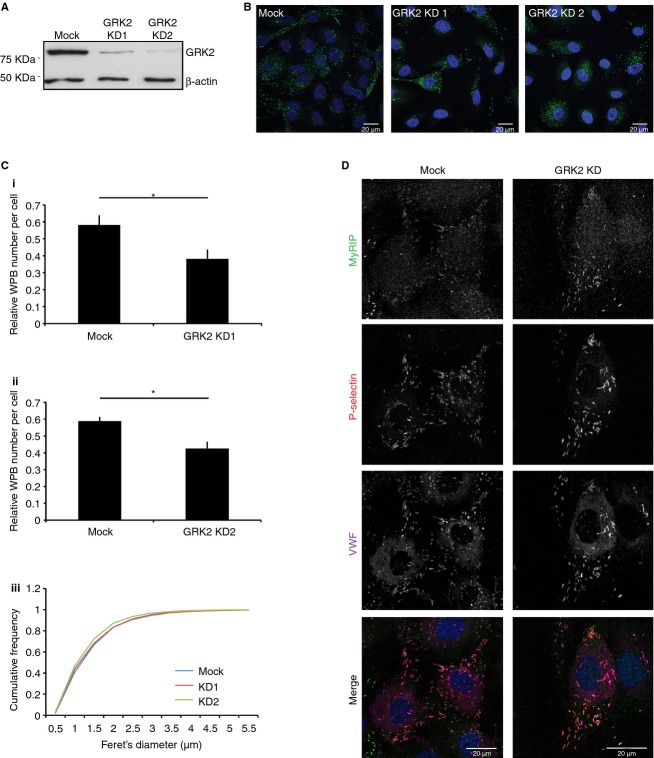
G protein-coupled receptor kinase 2 (GRK2) KD reduces WPB number without affecting biogenesis. (A–C) HUVECs were transfected with one of two siRNA sequences targeting GRK2, or with luciferase siRNA for mock treatments. (A) Representative western blot. GRK2 depletion was 85% (49–98%, *n* = 5) for siRNA sequence 1 and 94% (79–99%, *n* = 7) for siRNA sequence 2 as determined by SDS-PAGE and quantified by densitometry. GRK2 levels were normalized to β-actin protein expression. (B) Cells were fixed and stained for VWF (green) and DAPI (blue) and imaged at 63× objective (20–25 fields of view per condition, 450–650 cells) as confocal stacks ready for quantification. Presented images are maximum intensity projections. Scale bar 20 μm. (C) Quantification of experiments represented in B. WPB number per cell is reduced by 34% (*n* = 4, *P* = 0.024) and 28% (*n* = 4, *P* = 0.021) following GRK2 KD using siRNA (i) sequence 1 and (ii) sequence 2, respectively. Data are normalized against the maximum mock value for each experiment. Error bars represent SEM. Statistics were performed using a Student's *t*-test. (iii) Distribution of WPB Feret's diameter, represented as a cumulative frequency chart of the percentage of total organelles found in 0.5 μm bins. (D) HUVECs transfected with luciferase (mock) or GRK2 (sequence 2) targeting siRNA were fixed and stained for P selectin (red), VWF (magenta) and MyRIP (green). Scale bar 20 μm.

To investigate a potential role for GRK2 in modulating endothelial behaviour, we examined whether GRK2-depleted HUVECs produce normal WPBs, a key determinant of endothelial inflammatory function. GRK2 depletion was, on average, 85% (siRNA1, *n* = 6) or 94% (siRNA2, *n* = 7) effective using two different siRNA sequences (Fig. 1A). Transfected cells were thus processed for image analysis. We observed a 34% (siRNA1, 29–49%, *n* = 4) and 28% (siRNA2, 19–36%, *n* = 4) reduction in WPB numbers upon GRK2 depletion, without any consistent change in organelle length (Fig. 1C).

To assess whether other WPB characteristics were altered, recruitment of P-selectin and MyRIP to organelles was also analyzed by immunofluorescence. P-selectin is an integral membrane protein incorporated into WPBs at the *trans-*Golgi network (TGN) 31, whereas the Rab27A-MyRIP-MyoVA complex is recruited later and predominantly found on mature organelles 32. Incorporation of these proteins into WPBs is therefore indicative of successful progression through two independent stages of biogenesis. Both proteins were found to localize correctly in GRK2-depleted cells, indicating that cargo selection and maturation is unaffected (Fig. 1D). Furthermore, in both cell populations, 52% of cells showed P-selectin recruitment to WPBs, suggesting it is targeted equally efficiently (data not shown).

**Figure 2:**
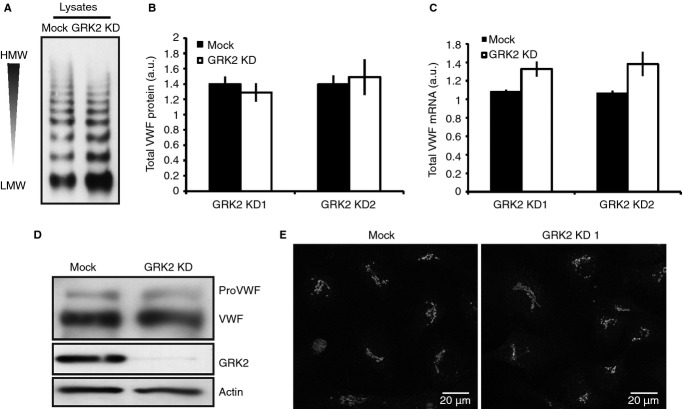
von Willebrand factor (VWF) synthesis and processing is normal in GRK2 KD cells. (A) Control and GRK2-depleted cell lysates were collected in serum-free medium and analyzed on 1.4% SDS-agarose gels under non-reducing conditions to separate VWF multimers. Representative immunoblot of VWF; multimerisation decreases from top to bottom. (B) VWF content in control and GRK2-depleted cell lysates was assayed by ELISA and normalized to total protein content as determined by BCA assay. To aid comparison between experiments, data were then normalized intra-experimentally to the maximal mock reading. Error bars represent SEM. There is no consistent change in VWF protein levels in GRK2 KD cells (*n* = 5). (C) Relative VWF mRNA expression in transfected cells was quantified by RT-qPCR. A consistent trend towards an increase in VWF expression of 24% (14–35%, *P* = 0.08) with siRNA sequence 1 and 31% (16–57%, *P* = 0.13) with sequence 2 is seen in GRK2 KD cells (*n* = 3). Statistics were performed using a Student's *t*-test. (D) Representative immunoblot. Mock and GRK2 KD cells were lysed in RIPA buffer and run on a 6% Tris-glycine gel to separate proVWF and VWF. Following transfer, the blot was cut and probed for VWF, GRK2 and β-actin. (E) Mock and GRK2 KD (siRNA sequence 2) cells were fixed and stained for TGN46 to label the TGN and imaged as confocal stacks at 40× objective. Images presented are representative regions of interest shown as maximum projections. Scale bars 20 μm.

Disruption of WPB biogenesis can result in the loss of higher molecular weight (HMW) forms of VWF 21. We therefore determined the multimeric state of intracellular VWF as an additional descriptor of organelle structure. VWF multimerisation was slightly reduced in GRK2-depleted cells, suggesting either VWF maturation is reduced or mature protein is being lost from the cell (see Fig. 2A).

### Reduction in WPB numbers is not due to reduced VWF expression

VWF drives the formation of WPBs 33. Deficiencies in VWF expression can therefore reduce WPB numbers, as seen in von Willebrand disease 34. To determine if this is causative of fewer organelles here, VWF protein levels in control and GRK2 knock-down (KD) cells were quantified by both ELISA and western blot and found to be comparable (Fig. 2). VWF transcript, however, was consistently, although statistically insignificantly, increased by 24% and 31% (siRNA1 and 2 respectively) in GRK2-depleted cells (Fig. 2C). Loss of GRK2 thus does not reduce WPB numbers by reducing VWF expression.

### VWF progression through the early secretory pathway is unaffected by GRK2 depletion

We next investigated whether WPB formation is GRK2 dependent. If WPB biogenesis is slowed, VWF is likely to accumulate in pre-Golgi compartments of the secretory pathway, as observed following expression of VWD-causing VWF variants 35. As VWF passage through the TGN is marked by the furin-mediated cleavage of its propeptide 36, VWF progression through the Golgi can be determined by the ratio of pro-VWF to VWF. As shown in Fig. 2(D), this ratio was unaffected by GRK2 depletion, as was the gross morphology of the TGN (Fig. 2E). GRK2 therefore does not influence WPB numbers by regulating VWF trafficking through the early secretory pathway.

### GRK2-deficient HUVECs secrete more VWF in the absence of secretagogue than control cells

**Figure 3:**
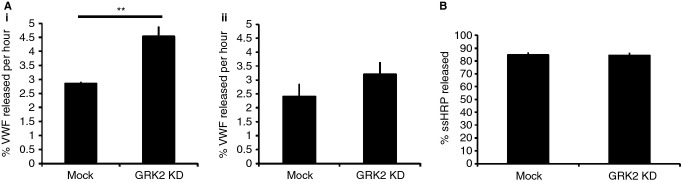
Unregulated secretion is increased in GRK2 KD cells. (A) Mock and GRK2 KD (siRNA sequence 2) cells were rinsed and incubated with (i) reduced-serum medium (optiMEM) for 7 h or (ii) serum-free medium for 4 h. Media and cell lysates were then collected and assayed for VWF content by ELISA, before normalizing to lysate total protein content as determined by BCA assay. The percentage of total VWF (medium plus lysates) released over 1 h was calculated and is presented here to allow comparison between the two conditions. Error bars represent SEM. Statistics were performed using Student's *t*-test. (Ai) A 59% increase in VWF release is seen in GRK2 KD cells assayed in OptiMEM (*n* = 3, *P* = 0.01). (Aii) In the absence of serum, a 33% increase in VWF secretion is seen (*n* = 4, *P* = 0.3). (B) HUVECs were transfected with luciferase- or GRK2-targeting siRNA plus ssHRP cDNA. Secreted ssHRP was collected over 17 h in phenol red-free medium before lysing cells in Tris-Cl pH2. The ssHRP content of media and lysates was determined by kinetic ELISA. Secreted ssHRP is expressed as a percentage of total ssHRP present in cells at the beginning of the assay (medium plus lysates) and is 84% under both conditions (82–88%, *n* = 3). Error bars represent SEM.

If VWF expression and WPB formation are normal in GRK2-depleted cells, reductions in organelle number are likely to be the result of increased WPB exocytosis or constitutive VWF secretion. Steady-state VWF release was therefore examined by ELISA. When incubated in reduced-serum medium in the absence of added secretagogue, GRK2-depleted cells released almost 60% more VWF than controls (Fig. 3Ai). Furthermore, a consistent (but statistically insignificant) 33% increase in unregulated secretion in the total absence of serum, and hence stimulus, was also seen in KD cells (Fig. 3Aii). GRK2-depleted cells thus possess fewer WPBs because they release more VWF under resting conditions. To determine whether constitutive secretion is up-regulated in GRK2-deficient cells, the secretion of ssHRP 28, a marker for this pathway, was monitored and found to be unchanged. GRK2 depletion therefore specifically affects VWF release (Fig. 3B).

### A GRK2-depleted endothelium is hyper-responsive to histamine stimulation

**Figure 4:**
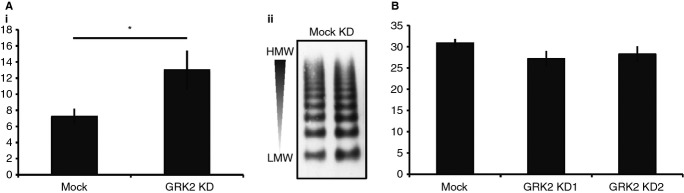
von Willebrand factor (VWF) secretion is increased in response to histamine but not PMA. (A–B) Mock and GRK2-depleted cells were incubated in serum-free medium plus (A) histamine for 30 min or (B) PMA for 45 min to monitor stimulated VWF secretion. Cells were lysed and the VWF content in all samples measured by ELISA and normalized to total protein content as determined by BCA assay. Secreted VWF is expressed as a percentage of the total intracellular VWF present at the start of the assay (releasates plus lysates). Error bars represent SEM. (Ai) Following histamine stimulation, VWF secretion is increased by 79% (*n* = 7, *P* = 0.03) in GRK2 KD cells. Statistics were performed using a Student's *t*-test. (Aii) Representative VWF multimer analysis. Histamine secretion assay samples were analyzed on 1.4% SDS-agarose gels under non-reducing conditions to separate VWF multimers and probed post-transfer for VWF. Multimerisation decreases top to bottom. (B) VWF secretion in response to PMA is unchanged following GRK2 depletion.

The observed change in VWF release, coupled to the known role of GRK2 in desensitization, suggested that WPB loss might result from enhanced sensitivity to GPCR signaling. To determine whether GRK2 depletion affects endothelial activation, we challenged cells with histamine, a GPCR agonist 37 and pro-inflammatory stimulant 38 of WPB exocytosis. GRK2 KD cells released 78% (45–179%, *n* = 6) more VWF than controls during a 30-min incubation with 10 μm histamine, consistent with increased sensitivity (Fig. 4A). There was no difference in the multimeric state of the VWF released by GRK2-deficient HUVECs, indicating that similar organelles are secreted in both cell populations (Fig. 4Aii).

We also performed a secretion assay in the presence of PMA, a membrane permeant DAG analogue, which stimulates WPB exocytosis by raising both intracellular calcium and cAMP levels, independent of cell surface receptors. Importantly, there was no difference in regulated secretion between control and KD cells in this instance, suggesting that the organelles of siRNA-treated cells are not generally secretion super-competent (Fig. 4B).

**Figure 5:**
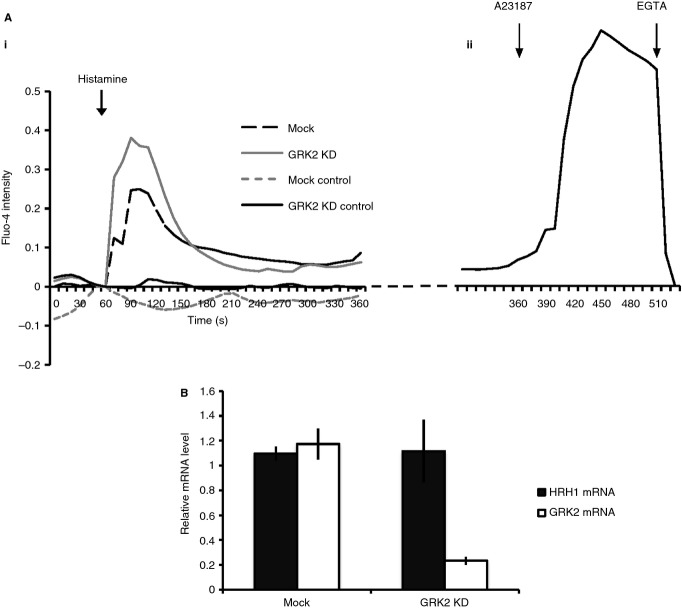
Signalling downstream of histamine is enhanced and prolonged in GRK2 KD cells. (A–B) Control and GRK2-depleted cells were loaded with Fluo-4 calcium indicator for 30 min then imaged by spinning disc microscopy, taking z stacks of 0.5-μm spacing every 10 s at 100× objective. Cells were treated with histamine, A23187 ionophore and EGTA after 1, 6 and 8 min of imaging, respectively. (A) Mean Fluo-4 intensity at each time-point imaged (three replicate experiments, in total 34 mock and 34 KD cells quantified) normalized against the maximum fluorescence seen in response to A23187 treatment to control for indicator loading. To allow comparison of cell responses, the intensity at time-point 0 is subtracted from all values to align curves. Unstimulated controls did not receive histamine treatment (six mock and 16 GRK2 KD cells). (ii) Mean curve for five mock cells from one replicate experiment to provide an example of Fluo-4 indicator response to A23187 and EGTA (*y*-axis as for Ai). (C) RNA was extracted from mock and GRK2 depleted cells and levels of HRH1 and GRK2 mRNA quantified by RT-PCR. Error bars represent standard deviation. There is no detectable change in HRH1 expression (*n* = 3).

To confirm that the enhanced histamine-stimulated secretion observed in GRK2-depleted cells was due to impaired GPCR desensitization, we next investigated the amplitude of downstream signaling events. Intracellular calcium concentrations before and during histamine stimulation were monitored by live-imaging cells loaded with Fluo-4 indicator. As shown in Fig. 5, Ca^2+^ influx in response to histamine was augmented in GRK2-depleted cells relative to controls. This was not due to general changes in receptor abundance, as HRH1 mRNA, the only histamine receptor expressed in HUVECs 39, was equivalent in GRK2 KD and control cells (Fig. 5B).

### Endothelial GRK2 is anti-inflammatory

**Figure 6:**
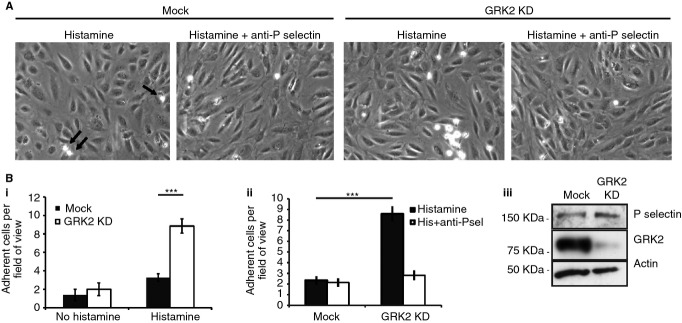
G protein-coupled receptor kinase 2(GRK2) depletion enhances THP-1 adherence to HUVEC monolayers. (A–B) Mock or GRK2-depleted HUVEC monolayers were stimulated with histamine and perfused with THP-1 cells under flow for 5 min in the presence or absence of P-selectin antibody. (A) Representative bright field images of HUVEC monolayers taken at the end of each assay. THP-1 cells can be distinguished as bright white circles; examples are highlighted by arrows. (B) Quantification of the number of THP-1 cells adhered to control and GRK2-depleted HUVECs after 5 min perfusion before or following histamine simulation. Values shown are the mean number of firmly adherent THP1 cells per field of view. Error bars represent SEM. Statistical significance was determined by Student's *t*-test. (Bi) The number of adherent THP-1 cells following stimulation is 170% higher on GRK2-depleted endothelial cells (*n* = 3, *P* = 1.94 × 10^−8^). (Bii) The increase inTHP-1 cells firmly adherent to GRK2-depleted HUVECs was blocked by the presence of anti-P-selectin (15 μg mL^−1^) by 67% (data from one experiment; mock, 2.33 ± 0.4; mock in the presence of anti-P-selectin, 2.12 ± 0.41; GRK2 KD, 8.58 ± 0.7; GRK2 KD in the presence of anti-P-selectin, 2.81 ± 0.47). THP-1 adherence to histamine-treated mock and GRK2 KD cells is significantly different, *P* = 1.4 × 10^−8^. (C) HUVECs transfected with luciferase or GRK2 siRNA were lysed and assayed for P selectin and GRK2 content by SDS-PAGE. As a control, blots were also probed for actin.

To establish the functional relevance of these changes, we assayed monocyte-like THP-1 cell adhesion to HUVEC monolayers. Control and GRK2-depleted HUVECs were stimulated with histamine before being perfused with 10^6^ THP-1 cells mL^−1^ under flow. The number of firmly adherent cells after 5 min was found to be increased almost 3-fold in GRK2-depleted cells (Fig. 6B). This increase was dependent on P-selectin, because, in the presence of 15 μg mL^−1^ function-blocking P-selectin antibody, THP-1 adhesion was reduced to control levels in GRK2 KD cells (Fig. 6B). There was no significant difference in adhesion between control and GRK2-depleted cells in the absence of secretagogue (Fig. 6B).

To verify that these observations result from increased WPB exocytosis, and not changes in P-selectin expression, P-selectin protein levels in mock and KD cell lysates were examined by SDS-PAGE and found to be comparable (Fig. 6C). Furthermore, P-selectin targeting to WPBs was not affected by loss of GRK2 (Fig. 1D). Increased THP-1 cell adhesion to GRK2-depleted HUVECs therefore results from increased delivery of P-selectin to the plasma membrane upon stimulation.

## Discussion

Extravasation of circulating leukocytes and their subsequent migration into afflicted tissue during inflammation must be tightly regulated, because excessive recruitment results in inflammatory disease. Leukocyte GRK2 activity is known to limit leukocyte migration along chemotactic gradients and thus prevent aberrant tissue infiltration 9,12,13,15,40. Here we show for the first time that GRK2 also regulates endothelial activation and leukocyte recruitment *in vitro*. Together these activities have the potential to limit both early and late events in the initiation of inflammation and thus prevent the harmful accumulation of leukocytes inside the body.

A key pro-inflammatory stimulant of endothelial cells is histamine 38, which triggers calcium-mediated WPB exocytosis by binding to HRH1 39,41, a GPCR phosphorylated by GRK2 37. Consistent with a failure in HRH1 desensitization, we report that histamine-invoked calcium influx is amplified in GRK2-deficient HUVECs, as is subsequent VWF release. Similar effects of GRK2 depletion on calcium signaling have been reported upon CCR5 stimulation in activated T cells, suggesting a general mechanism for regulation of inflammatory signal transduction 15. In addition to GRK2, HUVECs have been reported to express GRK5 and 6 42. To the best of our knowledge, there is no reported interaction between these kinases and HRH1 that may complicate interpretation of the results presented. GRK5 and 6 do, however, desensitize other receptors that are capable of stimulating WPB exocytosis, such as PAR-1 42, and thus may contribute to the regulation of other hemostatic and inflammatory pathways in a similar manner to GRK2.

Histamine signaling through HRH1 has previously been shown to induce P-selectin-mediated leukocyte rolling in post-capillary venules 39,43. Consistent with this, we see a 3-fold increase in the number of THP-1 cells adhering to GRK2-depleted HUVECs following enhanced receptor activation. These leukocyte-endothelial interactions rely on the regulated translocation of adhesion molecules and receptors to the cell surface. In the case of endothelial P-selectin, this is achieved through WPB exocytosis. The importance of P-selectin in mediating tethering between endothelial cells and leukocytes is well established 44. In P-selectin-deficient mice, both initial rolling, as seen by intra-vital microscopy, and subsequent neutrophil recruitment to inflamed sites are severely impaired 45. Similarly, antibodies against P-selectin glycoprotein ligand-1 (PSGL-1), the leukocyte counter-receptor for P-selectin, block rolling of human polymorphonuclear cells in rat mesenteric venules 46. Here we indicate that the converse is also true; enhanced delivery of P-selectin to the endothelial surface can promote excessive accumulation of adherent leukocytes.

Consistent with our data, increased cell-surface P-selectin has been shown to correlate with the development of inflammatory infiltrates in a number of pathologies. In murine atherosclerotic lesions, histochemical staining of endothelial P-selectin is strongest at sites of active macrophage infiltration 47. Moreover, VEGF-induced P-selectin up-regulation leads to psoriasis and contact dermatitis 48, whereas enhanced surface translocation of P-selectin in pancreatic capillaries, through WPB exocytosis, vastly contributes to the progression of severe acute necrotizing pancreatitis 49. Endothelial P-selectin expression is also increased in the inflamed synovial tissue of patients with rheumatoid arthritis 50, where it promotes monocyte-microvasculature interactions 51. Paradoxically, loss of P-selectin is reported to enhance progression of murine collagen-induced arthritis 52; the contribution of adhesion molecules to inflammatory responses is therefore complex. It is unknown whether endothelial GRK2 is down-regulated in any of these pathologies; however, it is tempting to speculate that augmentation of endothelial activation would serve to exacerbate the already enhanced chemotactic responses of GRK2-deficient leukocytes.

Recently, endothelial-targeted deletion of GRK2 in mice revealed that, even in the absence of pro-inflammatory mediators, loss of GRK2 in the endothelium triggers macrophage infiltration 16. This is attributed to up-regulation of cytokine expression in response to reactive oxygen species (ROS), generated by GRK2-depleted mitochondria. Here we provide a more direct mechanism by which loss of GRK2 could promote leukocyte adherence to the endothelial wall, and thus extravasation, through impaired inflammatory receptor desensitization and enhanced WPB exocytosis. This could occur downstream of the systemic cytokine signaling induced by loss of GRK2 itself, or upon development of inflammatory disease. How these mice respond to infection or induction of chronic inflammatory conditions remains to be tested.

The storage of both P-selectin and VWF in the same secretory compartment ensures coordinated delivery of both proteins to the cell surface, implying they function in the same processes. Although THP-1 accumulation in GRK2-depleted cells was P-selectin dependent here, the increased secretion of VWF from these cells may also affect inflammation. VWF is itself capable of interacting with PSGL-1 via its A1 domain, an interaction enhanced by the presence of β2-integrins 53. The involvement of the latter, plus observations of reduced rolling in P-selectin-deficient mice 45, suggests these interactions are more important in the latter stages of leukocyte recruitment and adhesion. The combined action of increased P-selectin and VWF secretion is therefore consistent with the strong impact of GRK2 depletion on firm adhesion, described here, as opposed to just rolling. We speculate that *in vivo*, the inflammatory effects of increased VWF secretion could be yet further enhanced through the recruitment of large numbers of activated platelets, which themselves induce P-selectin-dependent leukocyte rolling and WPB exocytosis 54. Platelets also up-regulate expression of additional adhesion molecules, such as E-selectin and VCAM-1 55, and further enhance leukocyte activation 56.

Delivery of a bolus of adhesion molecules to the endothelial membrane requires the formation of functionally competent secretory organelles. We find that GRK2 depletion does not significantly affect WPB morphology or cargo recruitment, including incorporation of P-selectin. Interestingly, loss of GRK2 does, however, result in a 30% reduction in WPB numbers. This may be explained by the observed increase in unregulated VWF secretion, which is most likely attributable to basal exocytosis of WPBs as described by Giblin *et al*. 57. Interestingly, this increase in VWF release is not accompanied by a change in steady-state levels of VWF protein, although as WPBs contain approximately 50% of all intracellular VWF 58, a 30% fall in organelle number is only expected to result in a relatively small reduction in total protein. As the relationship between levels of VWF and numbers of WPBs is complex, with both initial formation and subsequent pre-exocytic stages of WPB biogenesis offering opportunities to adjust this ratio 24, it is currently difficult to determine why a fall in protein is not observed.

The simplest explanation for the effects of GRK2 depletion on unregulated VWF secretion is a general failure in GPCR desensitization. The observed difference in VWF release between GRK2-depleted cells assayed in serum-free and reduced-serum media does suggest that unidentified extracellular agonists may play such a role. Unregulated secretion is, however, still enhanced by 30% in the absence of serum. This could result from autocrine or paracrine signaling; HUVEC confluency is known to affect WPB numbers 59. Alternatively, the recent observation that GRK2 depletion increases mitochondrial production of ROS 16, known stimulants of WPB exocytosis 60, may promote low-level endothelial-autonomous stimulation of WPB exocytosis.

Reduced organelle number had no inhibitory effect on histamine-evoked VWF secretion. On the basis that, at most, only 25% of total intracellular VWF was released from GRK2-deficient cells upon histamine stimulation, we suggest that organelle numbers are not limiting under these conditions. Whether WPB number is relevant under conditions such as chronic stimulation, remains to be determined. Moreover, *in vivo*, endothelial cells are unlikely to be confronted by a single stimulus following injury or infection. Angiotensin, vasopressin and adrenaline all bind to receptors that are under GRK2 regulation 61–63 and capable of stimulating WPB exocytosis 64–66. Indeed, VWF release is also enhanced in response to adrenaline in GRK-depleted HUVECs (data not shown). It may be that the combined activities of these secretagogues, enhanced by the absence of receptor desensitization, would exhaust WPBs in GRK-depleted cells quicker than in control cells.

In conclusion, our data are consistent with a model in which GRK2 limits endothelial activation at steady-state and during an inflammatory response by desensitizing GPCRs, including the histamine receptor, to the presence of agonist. Together with previously published data, this suggests that GRK2 activity could be required in both endothelial cells and leukocytes to limit excess cellular infiltration of inflamed tissues. The ability to modulate an inflammatory response in at least two of the cell types involved potentially allows for GRK2 to mount a coordinated control of this finely balanced process.

## Addendum

N. L. Stevenson performed research, carried out data interpretation and wrote the paper. B. Martin-Martin performed research. J. Freeman performed research. J. Kriston-Vizi performed research and contributed to design of the project. R. Ketteler contributed to design of the project. D. F. Cutler contributed to design of the project and interpretation of data, and wrote the paper.
